# An overview of the current evidences on the role of iron in colorectal cancer: a review

**DOI:** 10.3389/fonc.2025.1499094

**Published:** 2025-02-24

**Authors:** Mohammad Hasan Yousefi, Alireza Masoudi, Masoumeh Saberi Rounkian, Maryam Mansouri, Bita Hojat, Marzieh Kaveh Samani, Razieh Veisi, Parisa Honarvar Bakeshloo, Reza Nosratipour, Hamed Afkhami, Sepideh Saeb

**Affiliations:** ^1^ Department of Tissue Engineering and Applied Cell Sciences, School of Medicine, Qom University of Medical Sciences, Qom, Iran; ^2^ Cellular and Molecular Research Center, Qom University of Medical Sciences, Qom, Iran; ^3^ Department of Anesthesiology and Critical Care, Qom University of Medical Sciences, Qom, Iran; ^4^ Student Research Committee, School of Paramedicine, Guilan University of Medical Sciences, Rasht, Iran; ^5^ Student Research Committee, Shahrekord University of Medical Sciences, Shahrekord, Iran; ^6^ Student Research Committee, Kermanshah University of Medical Sciences, Kermanshah, Iran; ^7^ Department of Medical Microbiology, School of Medicine, Babol University of Medical Sciences, Babol, Iran; ^8^ Department of Biology, Islamic Azad University, Borujard, Iran; ^9^ Nervous System Stem Cells Research Center, Semnan University of Medical Sciences, Semnan, Iran; ^10^ Department of Medical Microbiology, Faculty of Medicine, Shahed University, Tehran, Iran; ^11^ Department of Allied Medicine, Qaen Faculty of Medical Sciences, Birjand University of Medical Sciences, Birjand, Iran

**Keywords:** colorectal cancer, iron metabolism, heme and non-heme iron, iron therapy, blood transfusion, iron deficiency, iron overload

## Abstract

Colorectal cancer (CRC) is a common and lethal malignancy that affects millions of people worldwide. Iron is an essential micronutrient that plays a vital role in various biological processes, but also has pro-oxidant and pro-inflammatory effects that may contribute to carcinogenesis. The relationship between iron and CRC is complex and influenced by multiple factors, such as dietary intake, absorption, storage, metabolism, and excretion of iron, as well as genetic and environmental factors that modulate iron homeostasis. This review article aims to provide an overview of the current evidences on the role of iron in CRC, discussing the potential mechanisms by which iron may affect CRC development and progression, as well as the implications for prevention and treatment. This review tries to focus on the following aspects: an introduction to iron and its role in CRC, role of heme and non-heme iron in CRC, dietary patterns, nutrition, and CRC, iron overload in CRC, iron deficiency and its role in CRC especially in surgery outcome and iron therapy and blood transfusion in CRC.

## Introduction

Cancer is a major global health challenge, affecting millions of people every year. According to the World Health Organization, cancer was the second leading cause of death worldwide in 2020, accounting for nearly 10 million deaths, or nearly one in six deaths (1, 2). Cancer is a malignant disease in which some of the body’s cells undergo uncontrollable division and tumor formation and may progress and spread to other parts of the body, called metastasis that can seriously damage normal tissues and organs (3, 4). Cancer can arise from various risk factors, such as genetic and epigenetic alterations, environmental exposures to different carcinogenic elements, infections and lifestyle behaviors (5, 6). It can also affect individuals of all ages, genders, races, and socioeconomic backgrounds, but some groups are more vulnerable than others due to biological, social, and environmental determinants of health (7). The prevention, diagnosis, treatment, and palliation of cancer are complex and multidisciplinary challenges that require coordinated efforts from researchers, clinicians, policymakers, and patients. Despite the considerable advances in different aspects of cancer researches, many gaps and barriers remain, especially in low- and middle-income countries, where the majority of cancer deaths occur (8–11).

Colorectal cancer (CRC) is a type of malignancy that affects the colon and/or rectum. It can be caused by various factors, such as genetic mutations, diet and lifestyle behaviors, infections, inflammation and sometimes without any known etiology. Regardless of the types of skin cancer, CRC is the fourth most prevalent cancer to be diagnosed globally and the second-greatest cause of cancer-related death (12–15). This malignancy is the second most common adult cancer in women and the third most common in men, and it is the fourth leading cause of cancer death, accounting for 9.2% of deaths worldwide (16, 17). The incidence of CRC varies depending on where it is found, with more cases occurring in developed nations and fewer cases in less developed ones.

Mortality from this malignancy varies by race. It is highest in non-Hispanic blacks and lowest in Asian Americans/Pacific Islanders. In recent years, the prevalence of this cancer has decreased in people over 50 years old, but it has increased in younger people (18). However, one of the characteristics of aging is hyperplasia, the most serious of which is cancer. Besides, cancer, like other aging-related diseases, mostly begins at the midpoint of life (19). In conclusion, the aging-related risk signature can predict the overall survival (OS), severity and immune cell infiltration of CRC patients (20).

Extensive CRC screening has substantially reduced incidence and mortality by enabling early detection and removal of precancerous adenomas (21). Multiple pervasive instigators may drive early-onset CRC (in patients <50-year-old), including global adoption of a westernized diet, chronic stress, and widespread use of antibiotics with alteration of the gut microbiota (22, 23). Environmental factors, such as western dietary habits, smoking, weight gain and obesity, diabetes, and heavy alcohol consumption, play major roles in causing sporadic CRC. Among several risk factors, the intestinal microbiota is an important contributor (24). Increasing evidence indicates that the intestinal microbiota plays a vital role in CRC initiation, progression, and metastasis (25). Intestinal microbiota dysbiosis alters host physiological functions, leading to various diseases (26, 27). Environmental factors and dietary habits are mostly responsible for the frequency of CRC (28). CRC development is known to be influenced by obesity and low physical activity and up to 70% of this cancer burden could be declined through improvement of dietary habits (29).

Evidence from twin and family studies indicates that only a small fraction of CRCs, including familial adenomatous polyposis (FAP), hereditary nonpolyposis colorectal cancer (HNPCC or Lynch syndrome), Peutz-Jeghers syndrome (a benign hamartomatous polyps in gastrointestinal tract), and other more rare disorders, are genetically predisposed (30, 31). Most of CRC cases are sporadic or non-inherited (32).

**Figure 1:**
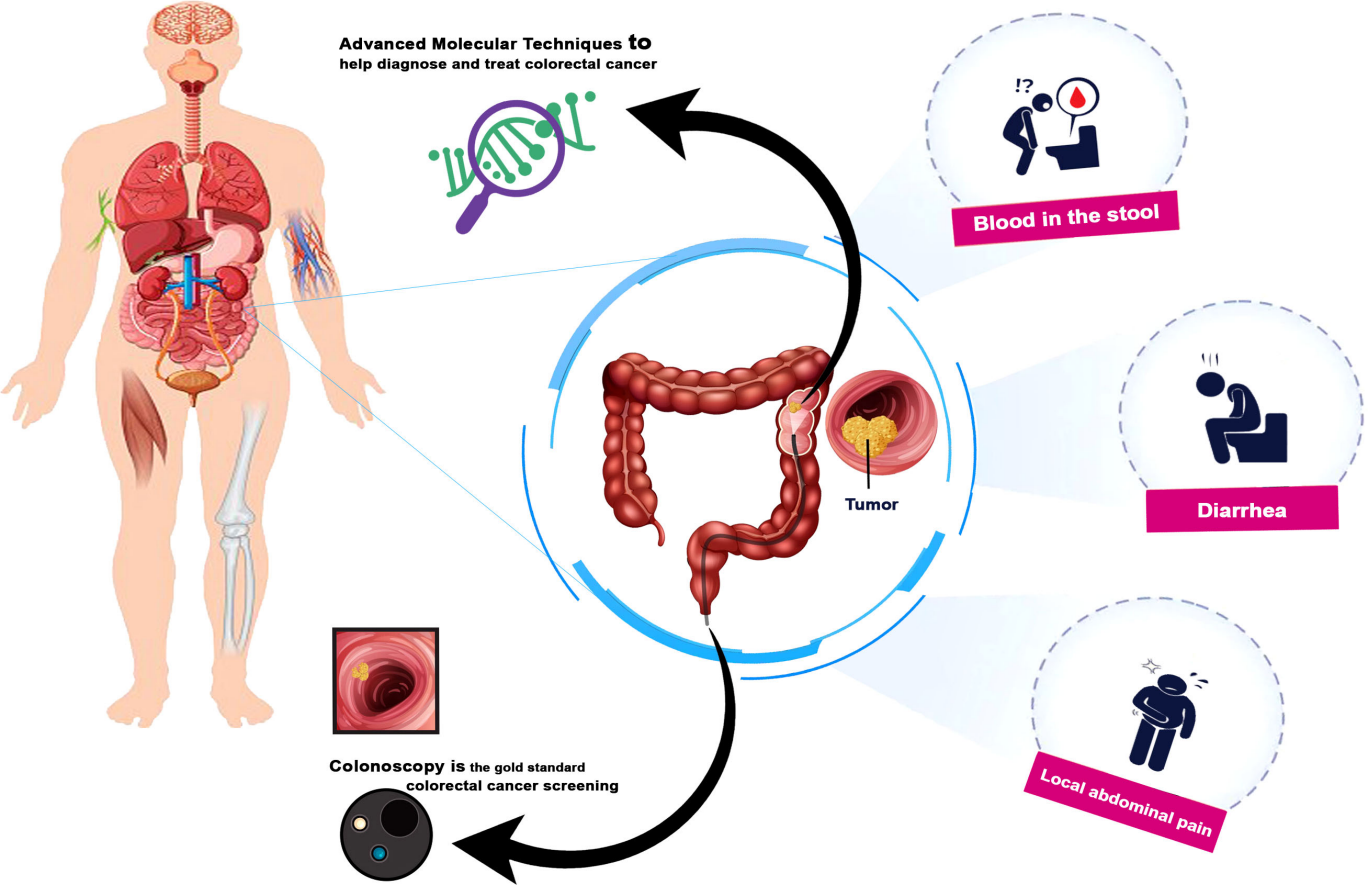
Colorectal cancer symptoms and Advanced diagnostic techniques.

Unfortunately, CRC clinical symptoms may not appear until the disease has already progressed, and even then, some people may be reluctant to seek medical attention. As a result of these delays, many CRC cases manifest at advanced stages and have a high death rate (33). As cancer grows, bowel habits change, including blood in the stool, diarrhea, alternating diarrhea and constipation, and local abdominal pain (34). The severe cases and mortality rate of colorectal cancer can be reduced by routine screening, early detection, and treatment of precancerous polyps and tumors (35). Colonoscopy and endoscopy are the gold standard for colorectal cancer screening, along with the development of advanced molecular techniques to help diagnose and treat colorectal cancer (36, 37) (**Figure 1**). Nowadays, colorectal mucus, urine, saliva and exhaled air are sources of additional diagnostic options/biomarkers (36, 38).

Iron (Fe), though, is an important trace element and a crucial micronutrient associated with several important biological processes. The ability of iron to transition through many oxidation states allows it to be utilized in a variety of physiological pathways such as cellular metabolism, respiration, also DNA replication and damage repair and several signaling pathways that are required to iron (39).

Iron bound to transferrin is the main source of iron for erythropoiesis, which enters the erythroblast by a process involving transferrin receptor-mediated endocytosis (40). This iron may be obtained through absorption of dietary iron and/or mobilization of iron stores in macrophages and liver (41). Iron export from macrophage and hepatocyte stores is also mainly carried out by ferroportin 1 (42).

When the iron is oxidized, released into the blood circulation, bound to transferrin and transferred to the places of use (40). The amount of iron required for the daily renewal of red blood cells (20-30 mg) is mostly through red blood cell iron recycling (41). Mainly, macrophages of reticuloendothelial system and duodenal enterocytes supply iron to the plasma (42). Therefore, since daily absorption (1-2 mg) only balances the daily loss, internal circulation of iron is necessary to meet bone marrow requirements for erythropoiesis (43).

Functional iron deficiency (FID), formerly known as anemia of chronic disease (44) is characterized by low serum iron and decreased transferrin saturation, in the presence of adequate body iron stores, as defined by the presence of dyeable iron in the bone marrow and/or serum ferritin levels within or above the normal range (45, 46). Finally, when there is decreased iron absorption and/or chronic blood loss, FID may convert to absolute iron deficiency (FID + ID) (47) for example in those presenting with low preoperative hemoglobin (Hb) (48).

Cancer patients may have absolute or functional iron deficiency as a result of the disease or its treatment (49). The use of oral iron formulations in patients with cancer and anemia is limited due to poor absorption in the duodenum, the need for high doses (three times a day), and the high possibility of gastrointestinal side effects (49–51). However, intravenous iron injection has significantly greater effects on hemoglobin levels and the measures of iron metabolism (49). We know that iron therapy may worsen tumor prognosis in patients with colorectal cancer by supporting colorectal tumor growth and increasing metastatic potential (50). This hypothesis is supported by *in vitro* studies, especially in CRC, that show the role of iron in all aspects of tumor growth. In addition, iron has been shown to enhance signaling in tumors harboring *APC* gene mutations, a mutation critical to the development of CRC. In one study, the current method of iron administration, as a treatment for anemia and as an alternative to blood transfusion, was considered dangerous and should be completely replaced. In this case, there are conflicts in the results of various articles. Some believe that the only concern about intravenous iron injection is anaphylactic shock, and that it is more effective than oral iron and iron supplements and increases the hemoglobin level. In another study, this uncertainty was more pronounced than the administration of intravenous iron, and it was stated that in anemic patients who require surgery for colorectal carcinoma, the current evidence is of insufficient quality to draw firm conclusions about the effectiveness of different measures for preoperative treatment (49, 50).

It has been demonstrated that iron metabolism has a pivotal and dual effect on both cell growth and death. Thus, it can play a key role in tumorigenesis and also design cancer treatment approaches (52–54). Iron related disorders such as the lack or low level of iron as well as iron overload can affect biological and molecular processes in the body that may be harmful, for example, in favor of carcinogenic changes such as colorectal cancer (55). Iron deficiency has the potential to result in an impaired immune response since hematopoiesis stem cells create all immune cells and iron is necessary for immune cell activity. This effect may lead to an altered tumor immunological microenvironment and a diminished immunosurveillance response, both which have the potential to hasten the development of the malignancy (12, 56). I this article, we tried to discuss different aspects of the role of iron in CRC including disease progression, prevention and treatment.

## Hemic and non-hemic iron in CRC

The vast majority of CRC or colon cancer cases are due to environmental causes rather than inherited genetic changes. Over the past decades, epidemiological evidence has demonstrated linking the consumption of red meat and, more convincingly, processed red meat with CRC. In parallel, hypotheses about the carcinogenic mechanisms of the consumption of red and processed red meat and the association with CRC have been proposed and investigated in biological studies (57).

**Figure 2:**
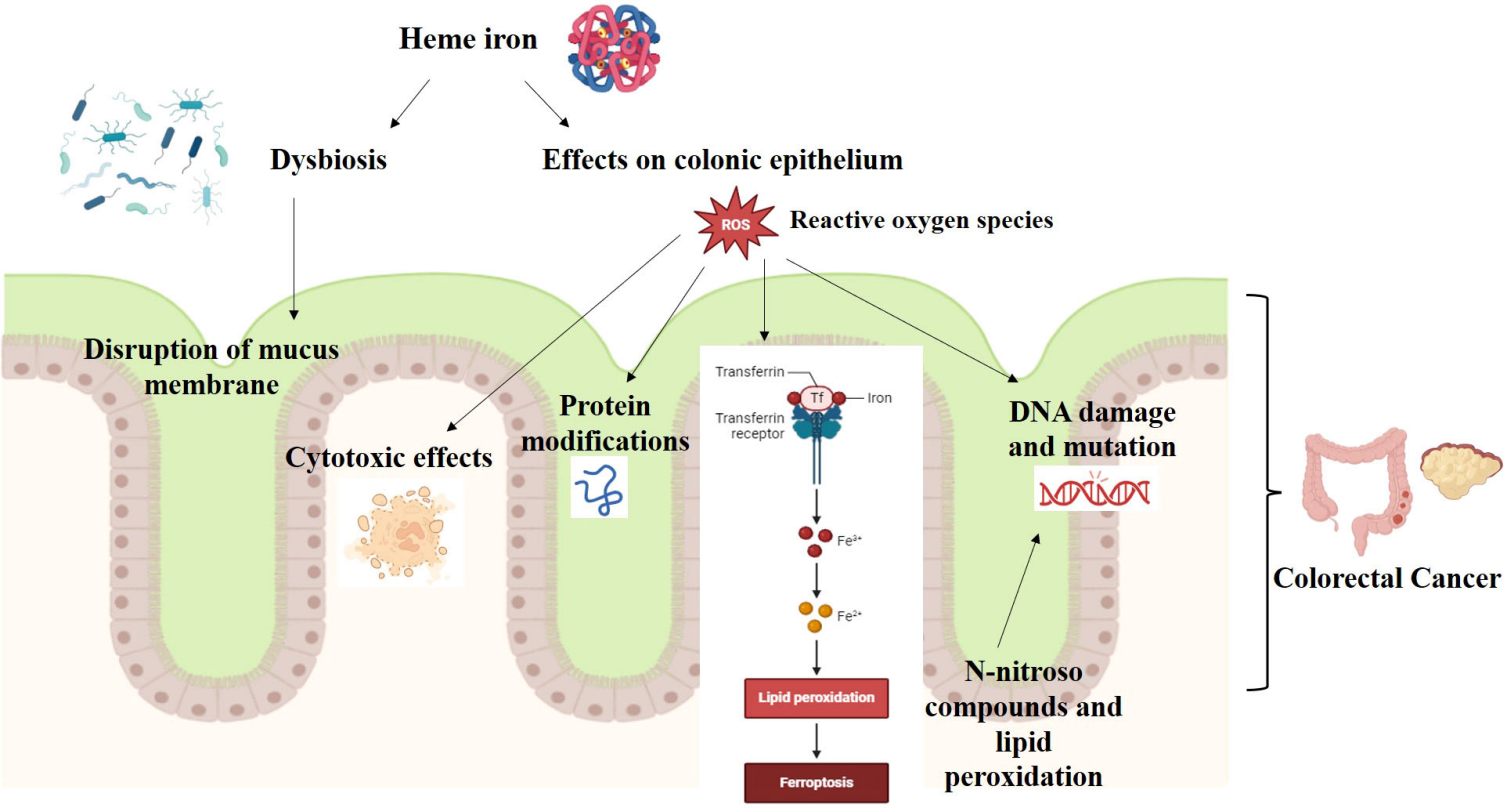
Heme iron and CRC carcinogenesis.

The presence of polycyclic aromatic hydrocarbons and heterocyclic aromatic amines, two groups of compounds known to be carcinogenic, has an additive (nitrosyl) effect on the formation of N-nitroso compounds and lipid peroxidation (13) (**Figure 2**). However, none of these hypotheses fully explain the association between red meat and processed red meat consumption and CRC risk. Consumption of red meat and processed meat is associated with the risk of developing colon cancer, one of the leading causes of death in rich countries.

Heme and nonheme iron are the two types of iron found in food from different sources. Nonheme iron is mainly found in plant-based foods, such as nuts, leafy green vegetables, and meat. Nonheme iron is also used to enrich flour. Red meat is the main source of heme iron, which is the central part of a porphyrin ring. Fish and poultry also contain some heme iron, but less than red meat. Most people get their iron intake from dietary supplements that include iron salts. Both forms of dietary iron can catalyze the production of free radicals and DNA damage, according to animal studies (56, 58) (**Figure 2**).

Both epidemiological and experimental evidences support the hypothesis that heme iron in meat increases the risk of colon cancer. In a meta-analysis study that included 566,607 individuals and 4,734 cases of colon cancer revealed that there is an association between dietary heme and colon cancer risk (59). Analysis of experimental studies on rat models of colon cancer induced by chemical carcinogens showed that dietary hemoglobin and red meat consistently increased foci of crypt abnormalities, a possible precancerous lesion. The mechanism is not clear, but heme iron has a catalytic effect on the endogenous formation of carcinogenic N-nitroso compounds and the formation of cytotoxic and genotoxic aldehydes by lipoperoxidation (59). Both pathways are involved in heme iron toxicity (29, 59). Heme iron, in particular, has been associated with increased colonic epithelial cell growth and promotion of CRC in rats (56, 58). Few studies have examined the association between heme iron intake and colorectal cancer risk (60). Heme iron, mainly derived from red meat, has been shown to have cytotoxic and hyperproliferative effects in the rat colon due to its higher bioavailability than non-heme iron (61). Heme iron can also generate reactive oxygen species, induce DNA damage, and modulate inflammatory and immune responses, which may contribute to colorectal carcinogenesis (62, 63).

However, some epidemiological studies have reported inconsistent results regarding the role of dietary iron and its heme and non-heme components in colorectal cancer development (59, 64). Therefore, more research is needed to clarify the complex interactions between iron metabolism and colorectal cancer. With regard to the risk of colorectal cancer in a Chinese population, Luo et al. studied several types and sources of Fe. Total dietary iron intake, non-heme iron intake, and iron from meat consumption were not significantly associated with the risk of colorectal cancer. According to this study, a Chinese population’s chance of developing colorectal cancer was increased by eating more heme and red meat and less iron through plants and white meat (65).

## Dietary patterns, nutrition and CRC

Over the past 15 years, there has been an increase in the utilization of dietary habits to examine how nutrition affects the risk of developing chronic diseases. Dietary patterns have grown in popularity as a thorough form of diet analysis that more accurately reflects the interactions between nutrients *in vivo* as they are ingested within a community. Dietary patterns are especially helpful for researching the relationship between nutrition and the onset of chronic diseases, since these conditions are frequently influenced by a number of interrelated factors that affect each other in different ways (66). Diet and lifestyle are important intervention targets in primary prevention because it has been predicted that dietary variables cause roughly half of all instances of colorectal cancer (67). Some variables are discussed here.

The gut microbiome plays a significant role in the development and progression of CRC. This microbiome consists of diverse and complex communities of microorganisms present in the human gastrointestinal tract, comprising over 10^14 bacteria and other microorganisms. The gut microbiome is involved in various physiological functions, including digestion, metabolism, immunity, and inflammation. However, it can also contribute to the pathogenesis of colorectal cancer due to dysbiosis (imbalance), which alters the gut environment, induces chronic inflammation, and affects the metabolism of dietary and endogenous compounds. Probiotics are live microorganisms that, when consumed in sufficient amounts, provide health benefits to the host. Probiotics can regulate the composition and function of the gut microbiome, as well as modulate the host’s immune system and metabolism. Therefore, probiotics have the potential for both preventing and treating colorectal cancer. They can restore gut microbiome balance, reduce inflammation, and regulate the bioavailability and activity of various compounds with anti-cancer effects (68).

Dietary iron is primarily divided into two categories: nonheme iron, which is present in plants, meat, nutrients, and heme iron, which is only present in meat. Iron salts as well as the iron mineral ferritin (FTN) are the two groups into which nonheme iron can be separated. Iron absorption is strictly controlled because of iron’s potentially harmful consequences. Iron has prooxidative properties that have the potential to stimulate the creation of reactive oxidative species that can harm DNA. The proliferation of colonic crypt cells is increased by dietary iron, and this in turn promotes the establishment of colorectal tumors, according to several rodent model studies. Additionally, it has been demonstrated that eating meat can raise the risk of developing colon cancer, and this impact is closely correlated with heme iron levels (69). The association between consumption of red or processed meat and CRC risk may be explained by a number of factors. First, high-temperature cooked beef contains carcinogenic heterocyclic amines. The intake of chicken is a major source of dietary heterocyclic amines, but it is not linked to the risk of cancer. The dosages of heterocyclic amines that cause cancer in animals are 1,000–100,000 times greater than the dose consumed by people. As a result, heterocyclic amines may not be a significant factor in the development of CRC. According to a second theory, the high saturated amount of red as well as processed meat raises the risk of CRC (70). Reduced rates of colorectal cancer are anticipated to result from public health initiatives that encourage moderate alcohol use, quitting smoking, weight loss, enhanced physical activity, and moderate eating of red meat (71). Additionally, Nitrite and nitrate compounds are found in foods exposed to nitrous oxide. Among these compounds, N-nitroso (NOC) can be mentioned. 20 NOC acts as a DNA alkylating agent and causes mutations in the genes responsible for cell proliferation and differentiation. Nitro reductase activity in intestinal bacteria increases if diets rich in meat and low in fiber are consumed. Fermentation products of red meat proteins such as ammonia, phenolic compounds, secondary bile acids, and hydrogen sulfide increase the risk of colon cancer (72). However, some studies could not find any significant association between iron intake and colon cancer. In 2007, Kabat et al. found that there was no association between iron intake, heme iron, or iron from meat and risk of colon cancer generally and this is not affected by alcohol use or hormone replacement medication (60). Ashmore et al. examined how dietary iron, heme iron, and additional iron affected the risk of colorectal cancer (CRC). It was found that intake of either heme iron or overall iron had no discernible links to the incidence of CRC. But, in contrast to men, women’s dietary iron consumption was negatively correlated with CRC. In both genders, a positive association between supplemental iron consumption of over 18 mg/day and CRC incidence was observed. These results imply that supplemental iron intake above 18 mg/day may raise the risk of CRC (58).

## Iron overload and CRC

**Figure 3:**
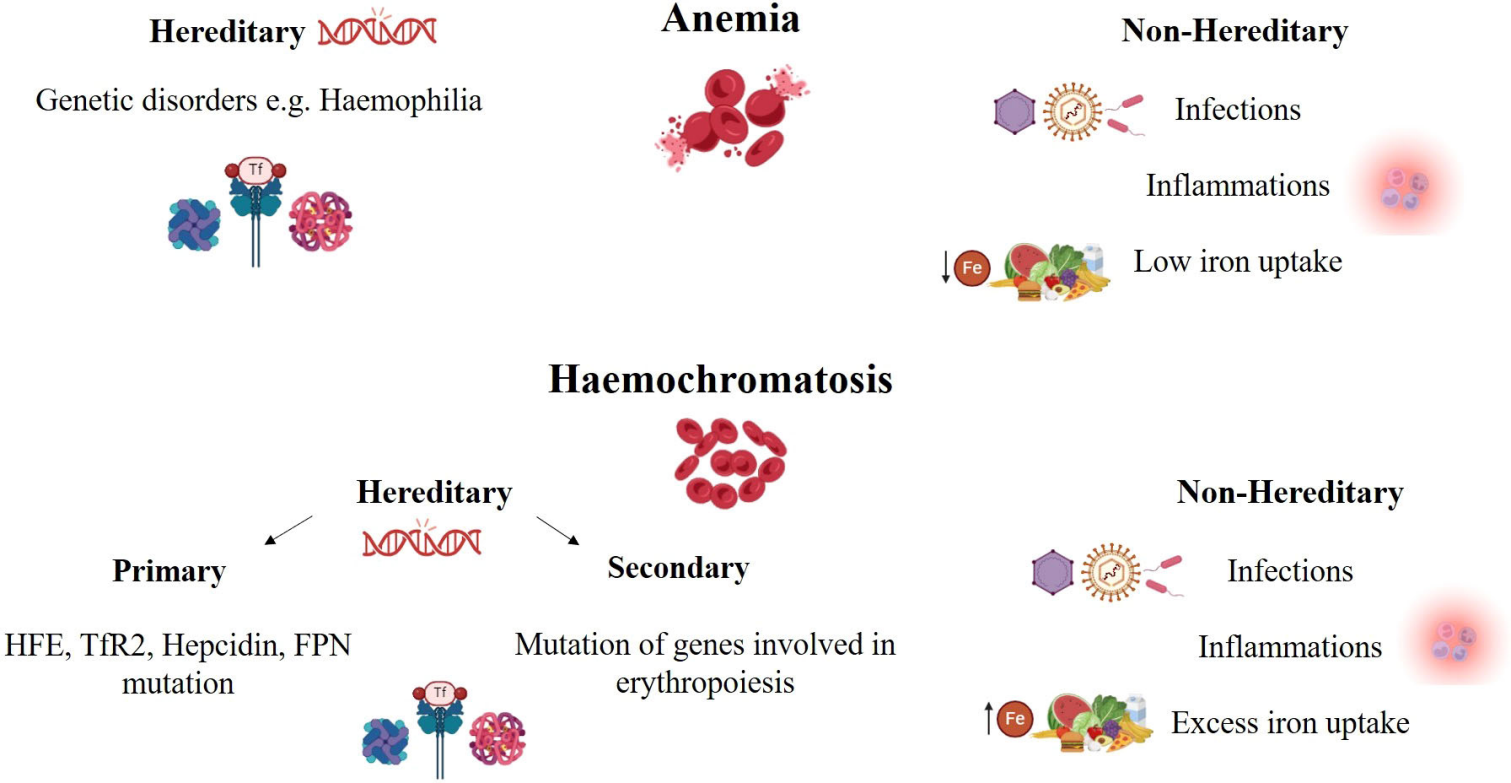
Iron deficiency and iron overloaded in CRC.

Both lack or low levels and overload of iron may have adverse effects on different aspects of CRC pathogenesis. The idea that too much iron raises the risk of colon cancer is supported by data, suggesting both dietary iron intake and body iron levels raise the risk of cancer (**Figure 3**). Even though numerous studies have thoroughly examined and analyzed the part that more iron plays in the development and spread of cancer, it hasn’t been proven to cause it yet (12).

Iron overload can have two unique sources; first, excess food iron (exogenous iron) that can enter colon tissue and affect it locally, straight forwardly from the lumen, and increased body iron resources and second, high serum level of iron (endogenous iron), that can harm target organs and cells. Most of the time, a normal person only absorbs 10% or less of the iron he consumes. As a result, a sizable portion of dietary iron is retained in the intestine without being absorbed and may pass into the colon (28). Controversial results were obtained from different researches conducted on the association of iron overload with CRC incidence and pathogenesis.

**Table 1** summarizes some studies about iron overload and the risk of different malignancies with a focus on gastrointestinal cancers.

**Table 1:** Some studies about iron overload and the risk of different malignancies especially CRC.

Study	Findings	Ref.
Females with colorectal adenoma and *HFE* gene mutation	Higher total body iron stores.	(73)
Homozygous p.C282Y or a compound heterozygous p.C282Y/p.H63D mutation	Increased the risk of developing HH and HCC, but not a significant increase in the risk of CRC and breast cancers.	(74)
Hemochromatosis	Increased the risk of esophageal squamous cell carcinoma and colon adenocarcinoma, but not of other gastrointestinal cancers.	(75)
HH mice model.	• Colitis and colon cancer progressed more strongly in *Hfe-/-* mice than in wild-type mice. • Sequencing of fecal 16S RNA showed considerable changes in the colonic microbiome in *Hfe-/-* mice: pathogenic bacteria like phyla Proteobacteria and TM7 were more dominant. • Expression of innate antimicrobial peptides declined • The level of pro-inflammatory cytokines increased significantly in *Hfe-/-* mouse colon.	(76)
Patients with advanced distal adenomas and two *HFE* mutations (C282Y and H63D), *HFE* polymorphism (IVS2 + 4) and the transferrin receptor gene polymorphism (G142S)	No association between any genotype and advanced adenoma, nor between dietary iron intake and adenoma risk or between *HFE* genotype and adenoma risk stratified by iron intake.	(77)
Individuals in the highest quintile of total iron binding capacity	• Significantly elevated risk of developing colorectal cancer.	(78)
Individuals with the *HFE* Tyr282 allele and homozygote for the TFR allele.	• Have a higher risk of breast cancer and CRC. • Increased affinity of transferrin receptor (TFR) for transferrin followed by increased cellular uptake of iron through complexes of the wild-type *HFE* gene product with the transferrin receptor and two *HFE* mutants (Cys282Tyr, His63Asp) may occur.	(79)
Patients with CRC were tested for the presence of the Cys282Tyr mutation	Heterozygosity for Cys282 HFE mutation: not seem to be a risk factor for colorectal carcinoma.	(80)
• Mutations in DNA mismatch repair genes (MMR)	• One of the corresponding factors for Hereditary nonpolyposis colorectal cancer (HNPCC).	(81, 82)
• Homozygosity for HFE H63D polymorphism	• May be a genetic modifier of disease manifestation and the risk of CRC. • Earlier onset of CRC in H63D homozygotes with a mean age of 6 years earlier. • The risk of CRC was significantly lower in women than in men regardless of *HFE* genotype.	

*HFE* gene encodes a protein involved in regulating hepcidin expression and iron absorption. Genetic mutations in this gene can lead to hereditary haemochromatosis (HH), a disorder that too much iron is absorbed from the diet (83). In a study, blood samples were collected from 36,826 women, among whom 527 were diagnosed with colorectal adenoma, and 527 matched controls who underwent endoscopy to rule out adenoma were identified. The relationship between HH and biochemical parameters of total body iron status, including transferrin saturation and the ratio of transferrin receptors to ferritin, were assessed. According to the results of this study, females with any *HFE* gene mutation had higher total body iron stores (73) (**Figure 3**).

It has been suggested that HH can increase the risk of some extrahepatic diseases such as CRC. In a study conducted on 3,645 Swedish patients with homozygous p.C282Y or a compound heterozygous p.C282Y/p.H63D mutation, risk of some disorders including hepatic manifestation such as cirrhosis and hepatocellular carcinoma (HCC) and extrahepatic diseases such as HH, breast cancer, CRC, type 1 and 2 diabetes, hypothyroidism, Parkinson’s disease and mortality were measured between 1997 and 2017. This study suggested that although these mutations increased the risk of developing HH and HCC, a significant increase in the risk of CRC and breast cancer was not observed (74).

According to another study that presents the results of a population-based cohort study of patients with hemochromatosis in Sweden from 1965 to 2013, the authors investigated the association between hemochromatosis and the risk of various types of gastrointestinal cancer. They found that hemochromatosis increased the risk of esophageal squamous cell carcinoma and colon adenocarcinoma, but not of other gastrointestinal cancers. They suggest that hemochromatosis might influence gastrointestinal carcinogenesis through iron overload and oxidative stress (75).

HH can disrupt colonic physiological interactions and promote colon inflammation and even cancer. *In vivo* researches that were done on HH model mice and wild mice, compared the development and severity of colitis and colon cancer, and the potential factors that contribute to these conditions.

The authors concluded that colitis and colon cancer progressed more strongly in *Hfe-/-* mice than in wild-type mice. The main and underlying reasons for this phenomenon are several factors that have been reported as follows: The crypt cells of mice with *Hfe-/-* genotype have lower proliferation capacity and cannot do the wound healing process efficiently, which drives inflammatory reactions following tissue injury. Also, it can lead to colon cells being leakier. About the host/microbiome interaction, Sequencing of fecal 16S RNA showed considerable changes in the colonic microbiome in *Hfe-/-* mice in such a way that pathogenic bacteria like phyla Proteobacteria and TM7 were more dominant and the number of bacteria adhering onto the mucosal surface of epithelium was increased. While the expression of innate antimicrobial peptides declined, the level of pro-inflammatory cytokines increased significantly in *Hfe-/-* mouse colon than in wild-type ones (76).

A study investigated whether heterozygosity for *HFE* mutations was associated with the risk of advanced distal adenoma and the possible role of dietary iron intake in this case. In several cancer screening trials including CRC, genotyping was performed for 679 patients with advanced distal adenomas and controls on two *HFE* mutations (C282Y and H63D), *HFE* polymorphism (IVS2 + 4) and the transferrin receptor gene polymorphism (G142S). The study found no association between any genotype and advanced adenoma, nor between dietary iron intake and adenoma risk or between *HFE* genotype and adenoma risk stratified by iron intake. These results do not confirm the relationship between *HFE* heterozygosity and the risk of advanced distal adenoma (77).

A review article about human studies investigated the association of iron with cancer risk. The evaluation has been done in the form of comparative studies, where people with and without colorectal neoplastic lesions were compared in terms of iron exposure. Almost three-quarters of the larger studies supported an association of iron, across all three strata, with the risk of neoplasia. The authors believe that since iron is a widespread supplement in the American diet, the benefits of iron supplementation are significant (84, 85). However, the risks of long-term exposure to iron, including a potential increase in the risk of colorectal cancer, need to be carefully measured (86).

In a research study, 226 cases of colorectal cancer were investigated alongside 437 matched references, focusing on a population from northern Sweden. The study findings revealed that individuals in the highest quintile of total iron binding capacity had a significantly elevated risk of developing colorectal cancer. But interestingly, the results indicated that high iron levels do not increase the risk of colorectal cancer, and HFE genotypes affecting iron absorption do not impact the risk of developing this type of cancer. However, one limitation of this study was occult bleeding from colorectal cancer, which could potentially impact iron markers (78).

It has been demonstrated that individuals with the HFE Tyr282 allele and homozygote for the TFR allele have a higher risk of breast and CRC. In the analysis of HFE Tyr homozygotes and compound heterozygotes, in combination with TFR serum homozygosity, the risk of various neoplastic disorders increased. Also, increased affinity of transferrin receptor (TFR) for transferrin followed by increased cellular uptake of iron through complexes of the wild-type HFE gene product with the transferrin receptor and two HFE mutants (Cys282Tyr, His63Asp) may occur. As a result of this study, it has been stated that the interaction between HFE and TFR alleles may increase the risk of various neoplastic disorders (79).

In another study, the DNA of 229 patients with CRC were tested for the presence of the Cys282Tyr mutation by digestion with Rsa1 and fragments separated by electrophoresis. Findings suggest that the heterozygosity for Cys282HFE mutation does not seem to be a risk factor for colorectal carcinoma (80).

Hereditary nonpolyposis colorectal cancer (HNPCC) is an autosomal dominant genetic disorder that can cause a type of CRC. Mutations in DNA mismatch repair genes (MMR) seem to be one of the corresponding factors for this malignancy (82). In a study, 362 individuals from Australia and Poland with confirmed MMR gene mutations were genotyped for C282Y and H63D polymorphisms to study the possible association of HNPCC disease phenotype and HFE gene polymorphisms. Based on the results of this study, the following findings were reported: Homozygosity for HFE H63D polymorphism may be a genetic modifier of disease manifestation and the risk of CRC was significantly higher for H63D homozygotes in comparison to the wild-type homozygotes and heterozygotes. There was also evidence for an earlier onset of CRC in H63D homozygotes with a mean age of 6 years earlier compared to wild-type or heterozygous participants. Between the two genders of Australian samples, the risk of CRC was significantly lower in women than in men regardless of HFE genotype for each of the single nucleotide polymorphisms. It can be concluded that understanding the mechanisms by which HFE interacts with colorectal malignancies could lead to a reduction in disease risk in HNPCC (81).

Dysmetabolic Iron Overload Syndrome (DIOS) refers to an increase in iron stores, when a certain cause of iron excess cannot be identified. DIOS is associated with metabolic syndrome and usually asymptomatic. But it can be diagnosed with metabolic syndrome components and steatosis. Histological investigations of liver tissue show iron overload. DIOS is considerably associated with oxidative stress and thus inflammation, diabetes, cardiovascular disorders and also cancer. Some studies indicate a relationship between high levels of serum ferritin and cancer. It seems that monitoring of iron and its related metabolites such as ferritin levels and transferrin saturation in serum of patients with metabolic syndrome, MAFLD (metabolic associated fat liver disease), cardiovascular disorders, NASH (nonalcoholic steatohepatitis) and so on is imperative, particularly in MAFLD and NASH, because iron deposition may lead to toxicity-mediated liver failure. Of course, more detailed studies of the role of DIOS in malignancies are still needed. Although DIOS findings are still controversial, it is clear that iron overload can disrupt cell functions due to ferroptosis (87).

One molecular evidence of the critical role of iron in oncogenicity is cancer cell’s resistance to ferroptosis. Ferroptosis is a type of cell death mediated by iron-dependent peroxidation of phospholipids controlled by several cell mechanisms. *In vitro* and *vivo* investigations show that oncogenic activation of PI3K-AKT-mTOR signaling pathway can confer resistance to ferroptosis in malignant cells. This requires activation of mTORC1(mammalian target of rapamycin complex 1) and mTORC1-mediated induction of SREBP1(sterol regulatory element-binding protein 1), an important transcriptional factor regulating lipid metabolism. Inhibition of SREBP1 or SCD1(stearoyl-CoA desaturase-1, a protein transcribed by SREBP1 activity, involved in the production of monounsaturated fatty acids) can sensitize cancer cells to ferroptosis. On the other hand, inducing expression of SREBP1 or SCD1 can restore ferroptosis resistance in cell line and mice models. However, further studies are necessary, especially in humans (88).

## Iron deficiency and CRC

Anemia is a frequent condition in patients with colorectal cancer (CRC), affecting about 40% of them (**Figure 3**). Anemia can lead to impair outcomes and quality of life of these patients and even patients’ survival (89, 90). According to previous studies, half of the CRC patients had iron deficiency anemia at the time of diagnosis. The iron stores in the body may be gradually reduced by hidden blood loss (13, 91). Iron deficiency anemia (IDA) is a common complication and a frequent presentation of colorectal cancer (CRC). When iron reserves are depleted, it is known as iron deficiency anemia in CRC patients. IDA results from chronic blood loss due to tumor bleeding, malabsorption of iron, or reduced dietary intake. Estimated total iron deficiency is done taking into account the amount of iron needed to restore a hemoglobin level of 13 g/dL and to replenish iron stores, as well as the estimated iron loss due to ongoing chronic bleeding and postoperative blood loss (92, 93).

IDA can cause symptoms such as fatigue, weakness, pallor, and dyspnea, which can impair the quality of life and prognosis of CRC patients (94). Inflammation brought on by cancer, intraluminal tumor bleeding, or a mix of both are thought to be the causes of iron shortage in CRC. It can be challenging to determine which factors are related to iron deficiency due to differences in nomenclature and diagnostic standards for the condition as well as tumor stage, tumor size, and tumor site (95). Prior studies have indicated that iron deficiency affects 60% of individuals with CRC. Additionally, chemotherapy drugs might cause anemia. Because it is inexpensive and convenient, oral iron supplements have historically been used to treat iron-deficiency anemia (IDA). Although almost 95% of oral iron is eliminated by CRC patients, oral iron therapy has been connected to a rise in gastrointestinal adverse effects (13, 94).

Iron deficiency, as well as iron overload, can both have potentially serious clinical repercussions for the development of cancer, hence iron consumption must be carefully managed. One of the therapeutic strategies for CRC is surgery. Nearly 90% of the total patients suffer anemia immediately following surgery. Preoperative anemia, perioperative excessive bleeding, inadequate nourishment during the postoperative period, and multiple blood samples for lab tests are the leading reasons. Additionally, increased hepcidin brought on by the body’s inflammatory reaction to surgery might prevent iron absorption and decrease iron release (43).

**Table 2** summarizes some studies about different preoperative iron therapy regimen and its impact on CRC patients after surgery.

**Table 2:** Preoperative iron therapy regimen and its impact on CRC patients after surgery.

Study	Findings	Ref.
Preoperative iron supplementation.	Confirmed the effectiveness of preoperative iron supplementation, reducing the need for blood transfusions and shortening hospital stays. Reducing the risk of anemia before surgery.	(96, 97, 98)
	No sufficient data supporting the significance of IVI supplementation following CRC surgery.	(99)
Administered iron sucrose (IS) at a dose of 600 mg intravenously to a group of anemic patients 2 weeks before undergoing colon cancer surgery.	No significant benefits in reducing LOS.	(100)
Compare two different iron therapy regimens; ferric carboxymaltose (FC) or iron sucrose (IS) in CRC patients after CRC surgery.	• An increase in the serum level of haemoglobin [Hb], reduce blood transfusion and length of hospital stay one month after the surgery in both iron therapy regimen. • Individuals receiving IS had a higher infection rate.	(101)
Two groups of colon cancer patients who received FC, IV and no-IV.	• Preoperative FC may be helpful for anemic patients who are iron deficient and undergoing surgery. • Reduced need for both perioperative and postoperative allogenic RBC transfusions as well as the time of hospitalization.	(102)
The prevalence of non-anemic iron deficiency in patients experiencing major surgery for CRC and its impact on the postoperative result.	Iron status parameters such as baseline ferritin, transferrin saturation and Hb were lower in deficient group, also experienced postoperative readmission and even more infections than iron-replete individuals.	(103)
Patients who underwent colon cancer surgery.	patients with untreated anemia stayed longer in the hospital.	(104)
Assessment of the effectiveness of preoperative intravenous iron therapy for treating colorectal cancer patients.	• IV iron could improve the Hb level of patients, • No considerable decrease in the proportion of patients requiring a postoperative blood transfusion.	(105)
Administration of 1000 mg of FC for intervention group VS. standard post-operative care for control group.	• Improved intermediate iron storage and quicker recovery of haemoglobin concentrations following surgery for the intervention group. • Received fewer allogeneic blood transfusions for the intervention group.	(106)

Anemia may play a role in complications before and after colon cancer surgery, such as higher death rates, lower quality of life, and higher length of hospital stays (LOS). One of the main risk factors for transfusion in surgery with moderate to large blood loss is a low preoperative hemoglobin concentration. The perioperative transfusions can have a negative impact on patient postoperative consequences (102). Postoperative results are linked with a LOS, upcoming challenges, and an increased chance of allogeneic blood transfusions (107). Transfusions can also be associated with some postoperative infections, CRC recurrence and mortality (101, 108, 109). The risk of morbidity and death in individuals undergoing major surgery is correlated with preoperative anemia and iron deficiency is the most typical cause of anemia in individuals having surgery for colorectal cancer. Findings from previous studies confirmed the effectiveness of preoperative iron supplementation, particularly with regard to reducing the need for blood transfusions and shortening hospital stays. These findings suggest iron deficiency is a desirable treatment target to at least improve short-term results (96, 97). However, there is no sufficient data supporting the significance of IVI supplementation following CRC surgery yet, which prevents some guidelines from recommending treatment until its advantages are established. However, due to ineffectiveness, guidelines do not advocate oral iron supplementation during the first few days following surgery too (99). But preoperative iron supplementation has gained attention as a potential strategy to improve outcomes by reducing the risk of anemia before surgery. Preoperative iron supplementation has been shown in some studies to reduce surgical transfusion and enhance short-term results (98).

A randomized clinical trial (RCT) was done by Edwards et al. and they administered iron sucrose (IS) at a dose of 600 mg or placebo intravenously to a group of anemic patients 2 weeks before undergoing colon cancer surgery that provided no significant benefits in reducing LOS (100). However, Further studies had different results. Administration of intravenous iron (IV) is a suggested regimen for anemia treatment after CRC surgery. Recently, a randomized, controlled trial has been done in order to compare two different iron therapy regimens; ferric carboxymaltose (FC) or iron sucrose (IS) in CRC patients after CRC surgery. In another study, it was mentioned that up to 1000 mg of iron can be given via FC in a single infusion and IS is limited to a daily dose of no more than 200 mg administered not over three times each week (110).

Overall, both of the supplied iron could lead to an increase in the serum level of haemoglobin [Hb], reduce blood transfusion and length of hospital stay one month after the surgery, but there was no significant difference between them. However, individuals receiving IS had a higher infection rate (101).

A similar study compared two groups of colon cancer patients who received FC, IV and no-IV. Calleja et al. concluded that preoperative FC may be helpful for anemic patients who are iron deficient and undergoing surgery. Also, the need for both perioperative and postoperative allogenic RBC transfusions was reduced dramatically by preoperative therapy, as well as the time of hospitalization (102).

Miles et al. in 2019 conducted a retrospective analysis of the prevalence of non-anemic iron deficiency in patients experiencing major surgery for colorectal cancer and its impact on the postoperative result. Patients were divided into two specific groups: iron-replete and iron deficient. In the deficient group, iron status parameters such as baseline ferritin, transferrin saturation and Hb were lower. Deficient patients also experienced postoperative readmission and even more infections than iron-replete individuals. So, the treatment of non-anemic iron deficiency before major surgery has been recommended (103).

Quinn et al. in 2021 gathered data from patients who underwent colon cancer surgery in a row to evaluate the impact of this process on patient outcomes and LOS. They concluded that patients with untreated anemia stayed longer in the hospital. The precise causes of these extended hospital stays are unknown, and they are likely caused by a variety of factors in addition to anemia. Anemia might be a sign of additional medical conditions that the patient has, which might delay their postoperative recovery (104).

Anemic patients also show a greater incidence of postoperative complications, and a higher likelihood of developing postoperative medical and surgical problems. It is crucial for clinicians to understand the value of routine screening and management of anemia, which can reduce these complications (111).

The findings of Wilson et al. contribute to establishing that preoperative intravenous (IV) iron therapy is effective for treating colorectal cancer patients and to the ongoing discussion about whether preoperative IV iron therapy improves postoperative outcomes. In this study, although, IV iron could improve the Hb level of patients, no considerable decrease was observed in the proportion of patients requiring a postoperative blood transfusion or experiencing postoperative challenges after the preoperative haemoglobin (Hb) level was optimized. Because this study is retrospective, more researches especially with prospective randomized trail nature, is strongly recommended for determining the short-and long-term advantages and disadvantages of IV iron (105).

Khalafallah et al. performed a randomized controlled trial. A maximal dose of 1000 mg of FC was administered over a 15-minute period for patients in the case group. The control group was given standard post-operative care, which included screening with blood transfusions as necessary. Improved intermediate iron storage and quicker recovery of haemoglobin concentrations following surgery were both related to intravenous FC. In comparison to the group receiving conventional treatment, the intervention group received fewer allogeneic blood transfusions. In their investigation, intravenous FC was shown to be a secure and reliable substitute for the treatment of postoperative functional iron deficiency anemia (106).

Perelman et al. in their systematic-meta-analysis realized that the quality of life and functioning outcomes focused on the patient were found to be unaffected by postoperative oral and intravenous iron treatments. However, no evidence was found in favor of the risk of adverse effects of oral iron, but proof of postoperative IV iron’s potential to shorten hospital stays should also be investigated more. In all patient groups undergoing elective surgery, the research could not find any evidence to recommend the usual utilization of postoperative iron supplementation (112).

Generally, it can be concluded that iron therapy has a considerable effect on the outcome of CRC surgery either as a pre-and post-operative intervention and should be applied carefully, and more researches are required for better understanding of the role of iron as a therapeutic strategy in different aspects of CRC.

## Iron therapy and blood transfusion in CRC

Anemia, usually due to iron deficiency, is very common in patients with colorectal cancer. Anemia is one of the most common extraintestinal manifestations of colorectal cancer (CRC) and may occur within 75% of patients. A study of 358 patients with CRC reported a 25% prevalence of moderate to severe anemia (Hb < 10 g/dL). Multivariate analysis showed that age, tumor location (right colon), and tumor size (large size), but not clinical stage or histological type, were significant influencing factors (43).

Inflammatory cytokines cause iron restriction in red blood cells, which reduces iron availability and impairs iron utilization. Allogeneic blood transfusion is widely used to treat anemia, but sometimes with poorer outcomes (113). Both anemia and allogeneic transfusions are associated with some adverse outcomes (114, 115).

Allogeneic red blood cell transfusions (ARBT) are associated with several risks, such as immunosuppression, transfusion reactions, and transmission of infections. However, the risk of these complications has been greatly reduced due to blood screening and new techniques for blood cells isolation. One of the most serious complications of ARBT is postoperative bacterial infection, which can increase morbidity, mortality, and costs (89, 116).

A recent study reported that ARBT was an independent risk factor for postoperative infection in CRC patients, with an odds ratio of 1.87 (89). Perioperative ARBT has been shown to adversely affect both short- and long-term outcomes of colorectal cancer (CRC) surgery (117).

Consequences of preoperative anemia is a major predictor for allogeneic blood transfusion (ABT) in surgeries with moderate to high intraoperative blood loss (48, 103).

In this context, ABT remains the most widely used treatment for acute perioperative and postoperative anemia, although its rapid and effective increase in Hb levels is transient (118). Postoperative ABT is associated with an increased rate of cancer recurrence (119). In a more recent meta-analysis, ABT has been shown to increase all-cause mortality, cancer-related mortality, and complications, such as wound infection, after CRC resection (120). The goal of treating anemia before surgery should be to normalize the haemoglobin level, according to the criteria of the World Health Organization (121). However, since CRC resections are procedures with moderate to high blood loss, achieving an Hb of 13 g/dL for both sexes is desirable to minimize the risk of transfusion (122). Munoz et al. developed evidence-based recommendations for perioperative and postoperative diagnosis and management of anemia and iron deficiency in surgical patients in order to achieve desired Hb levels that can prevent or reduce requirement of ABT (43). Sometimes Red blood cell transfusion (RBCT) used to treat anemia can be associated with significant postoperative risks and worse cancer outcomes and should be used based on evidences and patient’s condition (123, 124).

Management of anemia including iron therapy, in a multidisciplinary, multimodal and individualized strategy to minimize or eliminate allogeneic blood transfusion, has been shown to improve outcomes (125, 126). Iron therapy seems to improve manifestations of anemia related to CRC surgery. People who have low preoperative serum ferritin levels are more likely to have infections than those with normal levels (127). Normalization of haemoglobin level before surgery is recommended by the World Health Organization (41). Based on published evidence, oral iron is poorly tolerated and consumed. Intravenous iron is safe and effective (128), but is often avoided due to misinformation and misinterpretation of the prevalence and clinical nature of minor infusion reactions (129). In addition, some studies did not confirm its positive therapeutic effects. This may be due to the non-randomized design of the study (130–132). However, serious side effects are rare with intravenous iron (113). Intravenous iron is safe and effective, but is underused despite the very low risk of serious side effects (41, 129). Newer formulations of intravenous iron allow complete replacement of the dose in 15 to 60 minutes, which significantly facilitates care (46).

Hemoglobin change, 30-day postoperative complications and mortality, length of stay, and oncological outcomes are considered as the Secondary outcomes. One RCT and one non-randomized study (NRS) reported a reduction in the proportion of patients requiring RBCT using preoperative oral iron (43). Although it should be noted that RCTs were at high risk of bias and underpowered. One RCT on preoperative IV iron and one NRS on postoperative post-operative iron found no difference. Only one study showed a difference in the number of patients receiving IV iron. One RCT reported a significant increase in hemoglobin after this intervention. Among the 3 studies that reported length of stay, no differences were found. Although preliminary evidence suggests that this may be a promising strategy, there is insufficient evidence to support the routine use of intraoperative iron to reduce the need for RBCT in colorectal cancer surgery. Well-designed RCTs focusing on the need for RBCT and including long-term outcomes are required (133–135).

A systematic review tried to evaluate the effectiveness of preoperative iron supplementation in the treatment of anemia and its effect on the postoperative recovery of patients undergoing surgery for colorectal cancer by assessing 605 studies. Despite iron supplementation, three randomized controlled trials showed a decrease in hemoglobin levels. This was in contrast to four cohort studies, all of which showed a significant increase. All studies showed that transfusion rates were reduced following iron supplementation. None of the included studies evaluated postoperative complications. Due to heterogeneity in study design, treatment duration, doses, and variation in iron substrates, it was impossible to perform a meta-analysis. In anemic patients requiring surgery for colorectal carcinoma, the current evidence is of insufficient quality to draw firm conclusions about the effectiveness of different measures for the treatment of preoperative anemia (136).

## Conclusion

Colorectal cancer is one of the most common types of cancer in the world, and its occurrence is influenced by genetic and environmental factors. One of the environmental factors that is associated with an increased risk of this cancer is iron intake. Iron is an essential micronutrient that plays a role in many biological processes, but excess amounts of it can stimulate the growth of cancer cells, cause oxidative stress, and enhance oncogenic signals. Iron can be consumed as heme (found in red meat) or non-heme (found in vegetables and supplements), and both types of iron can increase the risk of colorectal cancer. On the other hand, iron deficiency and anemia can also be involved in the pathogenesis of colorectal cancer. Iron deficiency can impair the function of the immune system and alter the tumor microenvironment, which can both facilitate the development of cancer. In addition, iron deficiency can worsen the surgical and therapeutic outcomes for patients with colorectal cancer. Several studies were performed and different and sometimes controversial results were obtained. Therefore, a proper balance in iron intake and treatment is important for the prevention and management of colorectal cancer. In addition, team consensus and doing more experiments and monitoring based on every patient can help choosing the best treatment strategies and reduce adverse outcomes.
